# Association of the magnitude of the difference in blood pressure between office and ambulatory measurements with blood pressure variability in untreated individuals

**DOI:** 10.1186/s40885-022-00220-7

**Published:** 2022-12-15

**Authors:** Yea Je Lee, Moo-Yong Rhee, Je Sang Kim, Ungjeong Do, Ji-Hyun Kim, Byong-Kyu Kim, Hae-Young Kim

**Affiliations:** 1 Department of Internal Medicine, Dongguk University Ilsan Hospital, Goyang, Korea; 2 Cardiovascular Center, Dongguk University Ilsan Hospital, 27 Dongguk-ro, Ilsandong -gu, Goyang-si, Goyang, 10326 Gyeonggi-do Korea; 3 College of Medicine, Dongguk University, 123 Dongdae-ro, Gyeongju-si, Gyeongsangbuk-do 38066 Republic of Korea; 4 Cardiology Division, Department of Internal Medicine, Dongguk University Gyeongju Hospital, Gyeongju, Korea; 5 Department of Health Policy and Management, College of Health Science, Korea University, Seoul, Korea

**Keywords:** Blood pressure, Hypertension, Masked effect, Risk

## Abstract

**Objectives:**

We evaluated the association between cardiovascular risk factors and the magnitude of the difference in systolic blood pressure (SBP) between office and ambulatory measurements (masked effect) in untreated individuals without apparent hypertension-mediated organ damage (HMOD).

**Methods:**

The inclusion criteria were 1) age ≥ 20 years, 2) blood pressure ≥ 140/90 mmHg at the outpatient clinic, and 3) not receiving antihypertensive medications. The difference between office and ambulatory SBP was calculated by subtracting the ambulatory daytime SBP from the office SBP. The association between the masked effect and SBP variability was analyzed in individuals without HMOD (no electrocardiographic left ventricular hypertrophy, spot urine albumin-to-creatinine ratio < 30 mg/g, and estimated glomerular filtration rate ≥ 60 mL/min/1.73 m^2^, *n* = 296).

**Results:**

Among the cardiovascular risk factors, ambulatory BP variability was significantly correlated with the SBP difference. The standard deviation (SD) and coefficient of variation (cv) of 24-h SBP exhibited a significant negative linear association with the SBP difference in univariate and multivariate analyses adjusted for age, sex, presence of diabetes, and 24-h ambulatory SBP. A significant association was observed in patients with ambulatory daytime hypertension. In the multivariate analysis, individuals with a negative SBP difference > -5 mmHg exhibited a higher SD and cv of 24-h SBP than those with a negative SBP difference ≤ -5 mmHg or a positive SBP difference.

**Conclusions:**

The results of our study suggest that the magnitude of the negative difference in office and ambulatory SBP may be a potential risk factor, even in individuals without apparent HMOD.

**Trial registration:**

This trial is registered with ClinicalTrials.gov (NCT03855605).

## Introduction

Hypertension is an important risk factor for cardiovascular disease and is defined by using a cut-off threshold for office blood pressure (BP) or out-of-office BP. Although BP is a continuous variable, cut-off BP values are used for pragmatic reasons to simplify the diagnosis and decisions regarding treatment [1, 2]. However, the magnitude of BP elevation is important because meta-analyses of observational data have demonstrated a graded association of progressively increased cardiovascular risk from normal BP to elevated BP [3, 4], which is reflected in the classification and definition of the hypertension grade.

Among the hypertension phenotypes, masked hypertension is associated with a high cardiovascular risk that does not differ from that in patients with sustained hypertension [1, 2, 5]. Conventionally, masked hypertension is diagnosed using a combination of the pre-defined cut-off threshold of office BP and out-of-office BP [1, 2, 6, 7]. However, no studies have classified the grade of masked hypertension in relation to cardiovascular outcomes. A recent study reported that the magnitude of the negative difference between office BP and out-of-office BP (masked effect; higher daytime ambulatory BP than office BP) is associated with mortality in patients with chronic kidney disease but not in those with end-stage renal disease [8]. Similar to the association between the magnitude of BP elevation and cardiovascular risk, the magnitude of the masked effect may also be associated with the risk of cardiovascular disease. Therefore, the magnitude of the masked effect may be useful for classifying the severity of masked hypertension.

In the present study, we evaluated the association between the magnitude of the masked effect and cardiovascular risk factors in untreated individuals without apparent hypertension-mediated organ damage (HMOD).

## Methods

### Study population

From November 2015 to November 2019, 470 individuals who had high office BP (≥ 140/90 mmHg) at the outpatient clinic, who were not being managed with antihypertensive medications and required a diagnosis of hypertension, and who were at least 20 years of age were prospectively enrolled in the study. The primary study was designed to validate the diagnostic algorithm for hypertension using home and ambulatory BP measurements [9]. Individuals with secondary hypertension, hypertensive emergency or urgency, heart failure (New York Heart Association class III and IV), clinically significant cardiac arrhythmia, impaired renal function (serum creatinine ≥ 1.7 mg/dL), pregnancy, and history of drug or alcohol abuse within 6 months were excluded from the study. Those participating in night labor or shift work, participating in other clinical studies, having used other clinical trial drugs within the past month, or using drugs known to affect BP (e.g., steroids, monoamine oxidase inhibitors, oral contraceptives, or sympathomimetics) were also excluded. The study protocols and informed consent form were reviewed and approved by the Dongguk University Ilsan Hospital Institutional Review Board (No. 2015–102). Written informed consent was obtained from all participants before their entry into the study. Among the participants, the data of individuals without electrocardiographic left ventricular hypertrophy (LVH) and/or hypertension-induced renal damage were included in the analysis of this study.

### Measurements of office and ambulatory BP

The BP measurement schedule and study protocols have been described elsewhere [9, 10]. Office BP was measured by the study nurses. Participants were asked to avoid smoking, caffeine-containing beverages, and exercise within 30 min preceding the measurements. After 5 min of seated rest, three readings of office BP were obtained at 1-min intervals at each visit using an appropriate cuff size. The BP of both arms was measured simultaneously using WatchBP Office (Microlife, Taiwan). The office BP readings obtained every three visits were averaged, and the BP of the arm with the higher average BP was used as the office BP of the index arm.

At the second visit, ambulatory BP monitoring over 25 h was performed on the non-dominant arm using an automated, noninvasive oscillometric device (Mobil-O-Graph, I.E.M GmbH, Germany) with a measurement interval of 30 min. The participants were instructed to continue their normal daily activities during the day. A valid measurement was defined as valid readings for more than 70% of the total measurement attempts, at least 14 measurements during the daytime (09:00 to 21:00) and at least seven measurements during the nighttime (00:00 to 06:00 h).

Blood samples for hematologic and biochemical analyses were obtained after at least 8 h of overnight fasting.

### Definition of hypertension by office BP and ambulatory BP

Average daytime ambulatory BP was used as the reference standard for the diagnosis of hypertension. An average daytime systolic BP (SBP) ≥ 135 mmHg and/or diastolic BP (DBP) ≥ 85 mmHg was defined as ambulatory daytime hypertension. Office hypertension was defined as an office SBP ≥ 140 mmHg and/or DBP ≥ 90 mmHg in the index arm.

Systolic hypertension phenotypes were classified into systolic normotension (office systolic and daytime ambulatory systolic normotension), white-coat systolic hypertension (office systolic hypertension but daytime ambulatory systolic normotension), masked systolic hypertension (office systolic normotension but daytime ambulatory systolic hypertension), and sustained systolic hypertension (office systolic and daytime ambulatory systolic hypertension).

### Assessment of asymptomatic hypertension-mediated organ damage

Basic assessments of HMOD were performed based on the presence of 12-lead electrocardiographic LVH, urine albumin-to-creatinine ratio (ACR), and estimated glomerular filtration rate (eGFR) [1]. LVH was defined based on Sokolow-Lyon criteria (voltage of S wave in V1 + voltage of R wave in V5 or V6 > 35 mm or R wave in aVL ≥ 11 mm). Hypertension-induced renal damage was defined as eGFR < 60 mL/min/1.73 m^2^, calculated using the 2009 CKD-Epidemiology Creatinine formula [11], or spot urine ACR ≥ 30 mg/g.

### Statistical analysis

Continuous variables are expressed as the mean ± standard deviation, and categorical variables are expressed as numbers (percentages). SBP differences were calculated by subtracting the ambulatory daytime SBP from the office SBP. SBP variability was obtained based on the standard deviation (SD) and coefficient of variation (cv) of the averaged ambulatory BP. The cv was calculated by dividing the SD by the mean SBP. The average real variability (ARV) of ambulatory SBP was calculated using the suggested formula [12].

The correlations between SBP differences and the following cardiovascular risk factors were analyzed: age; sex; body mass index; low-density lipoprotein (LDL) cholesterol level; fasting blood glucose level; HbA1C level; eGFR; ACR; SD of 24-h, daytime, and nighttime SBP; cv of 24-h, daytime and nighttime SBP; and ARV of 24-h SBP.

Cardiovascular risk factors that had a significant correlation with the SBP difference were selected, and linear regression analysis was performed to demonstrate the relationship between the SBP difference and selected cardiovascular risk factors. Variables showing significant associations with the SBP difference in the univariate linear regression analysis were entered into the stepwise multivariate regression model and adjusted for age, sex, presence of diabetes, and ambulatory SBP; SD, cv, and ARV of 24-h SBP were adjusted for 24-h SBP, SD of daytime SBP for daytime SBP, and SD of nighttime SBP for nighttime SBP.

Differences between office and daytime ambulatory SBP were categorized as follows: negative SBP difference (masked effect; ME) > –5 mmHg, ME –5 to < 0 mm Hg, positive SBP difference 0 to < 5 mm Hg (reference; Ref group), positive SBP difference (white-coat effect; WE) 5 to < 10 mm Hg, and WE ≥ 10 mm Hg [8]. The SBP variabilities between the categorized groups and hypertension phenotypes were compared using an analysis of covariance adjusted for age, sex, presence of diabetes, and ambulatory SBP.

Differences were considered statistically significant at two-sided *p*-values < 0.05. Analyses were performed using SPSS (version 24.0; IBM Co., Armonk, NY, USA) and MedCalc Version 19.0 (MedCalc Software Ltd., Ostend, Belgium).

## Results

Of the 470 participants, the data of 296 participants who had valid 24-h ambulatory blood pressure monitoring data and did not have HMOD were included in the analysis. Table 1 shows the demographic and clinical characteristics of the study participants. The mean age was 51.6 ± 10.2 years, and 134 (45.3%) participants were men. Hypertension was observed in 69.9% of the study participants (*n* = 207) based on office BP and in 80.7% of the participants (*n* = 239) based on ambulatory daytime BP.

**Table 1:** Baseline clinical and demographic characteristics of study population

**Variables**	
n	296
Sex	
Male, n (%)	134 (45.3)
Female, n (%)	162 (54.7)
Age, years	51.6 ± 10.2
Body mass index, kg/m^2^	25.3 ± 3.4
eGFR, ml/min/1.73 m^2^	99.2 ± 12.4
spot urine ACR	9.3 ± 6.6
LDL cholesterol, mg/dL	132.8 ± 32.5
Fasting blood glucose, mg/dL	103.5 ± 19.9
HbA1C, %	5.7 ± 0.6
Diabetes, n (%)	36 (8.8)
Office hypertension, n (%)	207 (69.9)
Ambulatory daytime hypertension, n (%)	239 (80.7)
Office SBP, mmHg	140.5 ± 9.1
Office DBP, mmHg	91.1 ± 8.1
24-h ambulatory SBP, mmHg	133.3 ± 11.5
24-h ambulatory DBP, mmHg	88.2 ± 10.3
Daytime ambulatory SBP, mmHg	137.9 ± 12.3
Daytime ambulatory DBP, mmHg	92.0 ± 11.0
Nighttime ambulatory SBP, mmHg	124.7 ± 13.6
Nighttime ambulatory DBP, mmHg	81.0 ± 11.1

*eGFR* Estimated glomerular filtration rate, *ACR* Albumin to creatinine ratio, *LDL* Low density lipoprotein, *SBP* systolic blood pressure, *DBP* Diastolic blood pressure

The eGFR values were calculated using the 2009 CKD-Epidemiology creatinine equation. Systolic hypertension phenotype was defined by the level of office and ambulatory daytime systolic blood pressure

The SBP difference exhibited significant negative correlations with eGFR, 24-h SBP, daytime SBP, nighttime SBP, SD of 24-h SBP, SD of nighttime SBP, cv of 24-h SBP, and ARV of 24-h SBP and positive correlations with age and office SBP (Table 2).

**Table 2:** Correlation analysis between systolic blood pressure difference and cardiovascular risk variables

Variables	Correlation coefficient	*P* value
eGFR	-0.130	0.026
LDL cholesterol	0.066	0.261
Glucose	-0.030	0.604
HbA1C	-0.074	0.227
Spot urine ACR	-0.089	0.137
BMI	-0.112	0.054
Age	0.207	< 0.001
Office SBP	0.250	< 0.001
24-h SBP	-0.556	< 0.001
Daytime SBP	-0.697	< 0.001
Nighttime SBP	-0.293	< 0.001
SD of 24-h SBP	-0.358	< 0.001
SD of daytime SBP	-0.112	0.054
SD of nighttime SBP	-0.147	0.011
cv of 24-h SBP	-0.197	0.001
cv of daytime SBP	0.081	0.166
cv of nighttime SBP	-0.070	0.231
ARV of 24-h SBP	-0.184	0.001

Systolic blood pressure difference (office systolic blood pressure – ambulatory daytime systolic blood pressure)

*eGFR* Estimated glomerular filtration rate, *LDL* Low density lipoprotein, *ACR* Albumin-to-creatinine ratio, *BMI* Body mass index, *SBP* Systolic blood pressure, *SD* Standard deviation, *cv* Coefficient of variation, *ARV* Average real variability

**Fig. 1:**
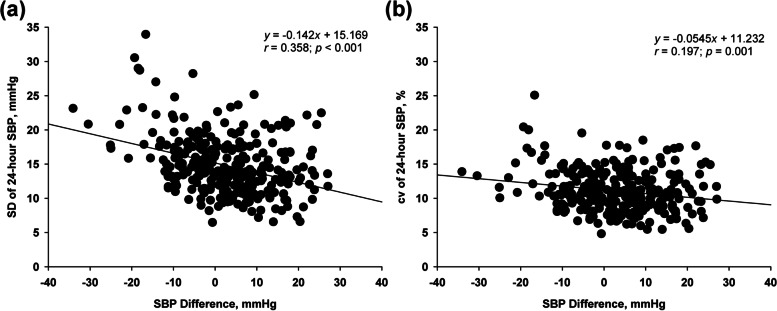
The association of SBP difference with SBP variability parameters. SBP difference was negatively associated with (**a**) SD of 24-h SBP and (**b**) cv of 24-h SBP. SBP, systolic blood pressure; SD, standard deviation; cv, coefficient of variation

Among the parameters of SBP variability, the SD of 24-h and nighttime SBP, cv of 24-h SBP, and ARV of 24-h SBP exhibited significant negative linear associations with the SBP difference. In the multivariate linear regression analysis adjusted for age, sex, presence of diabetes, and ambulatory SBP, the associations between SBP difference and the SD and cv of 24-h SBP remained significant (Table 3, Fig. 1). In the subgroup analysis of patients with ambulatory daytime hypertension, the negative linear association of the SBP difference with the SD and cv of 24-h SBP remained significant (Table 3). The eGFR was not associated with the SBP difference in the multivariate analysis.

**Table 3:** Univariate and stepwise multivariate linear regression analysis for the association of systolic blood pressure difference with blood pressure variability parameters

Variables	Univariate		Multivariate	
	**Beta (95% CI)**	***P* value**	**Beta (95% CI)**	***P* value**
All				
SD of 24-h SBP	-0.142 (-0.185 to -0.100)	< 0.001	-0.115 (-0.164 to -0.065)	< 0.001
SD of daytime SBP	-0.048 (-0.096 to 0.001)	0.054	Not selected	
SD of nighttime SBP	-0.058 (-0.103 to -0.013)	0.011	-	
cv of 24-h SBP	-0.055 (-0.086 to -0.023)	0.001	-0.070 (-0.100 to -0.039)	< 0.001
cv of daytime SBP	0.024 (-0.010 to 0.057)	0.166	Not selected	
cv of nighttime SBP	-0.012 (-0.055 to 0.013)	0.231	Not selected	
ARV of 24-h SBP	-0.061 (-0.098 to -0.024)	0.001	-	
Ambulatory daytime hypertension				
SD of 24-h SBP	-0.155 (-0.206 to -0.104)	< 0.001	-0.126 (-0.182 to -0.070)	< 0.001
SD of daytime SBP	-0.063 (-0.120 to -0.006)	0.030	-	
SD of nighttime SBP	-0.057 (-0.110 to -0.005)	0.032	-	
cv of 24-h SBP	-0.070 (-0.107 to -0.034)	< 0.001	-0.078 (-0.114 to -0.042)	0.001
cv of daytime SBP	0.004 (-0.034 to 0.041)	0.850	Not selected	
cv of nighttime SBP	-0.029 (-0.068 to 0.010)	0.149	Not selected	
ARV of 24-h SBP	-0.070 (-0.114 to -0.025)	0.002	-	

Systolic blood pressure difference (office systolic blood pressure – ambulatory daytime systolic blood pressure)

Dashes indicates that the variables were not included in the multivariate stepwise linear regression model

Multivariate stepwise linear regression analysis was adjusted with age, sex, presence of diabetes and blood pressure (SD, cv and ARV of 24-h SBP with 24-h SBP; SD of daytime SBP with daytime SBP; SD of nighttime SBP with nighttime SBP)

*95% CI* 95% confidence interval, *SBP* Systolic blood pressure, *DBP* Diastolic blood pressure, *SD* Standard deviation, *cv* Coefficient of variation, *ARV* Average real variability

The 24-h and daytime SBP values were higher in the ME > –5 mmHg group than in the ME –5 to < 0 mm Hg, Ref, and WE groups. Individuals with ME > –5 mmHg also had higher nighttime SBP than those with WE ≥ 10 mmHg (*p* < 0.05). The ME –5 to < 0 mmHg group had higher daytime SBP than the Ref and WE groups. There were no differences in BMI, eGFR, fasting blood glucose, LDL cholesterol, HbA1C, and urine ACR among the groups (Table 4).

**Table 4:** Demographic and clinical characteristic of five groups categorized by systolic blood pressure difference

	**Systolic blood pressure difference between office and daytime ambulatory blood pressure**					
	**Masked effect**		**Reference**	**White-coat effect**		
	** > -5 mmHg**	**-5 to < 0 mmHg**	**0 to < 5 mmHg**	**5 to < 10 mmHg**	** ≥ 10 mmHg**	p*
n	70	52	50	53	71	
Age, years	50.5 ± 8.7^a,b^	49.4 ± 9.6^a^	50.3 ± 9.9^a^	51.3 ± 12.9^a,b^	55.5 ± 9.1^b^	0.005
Sex						
Male (%)	34 (48.6)	26 (50.0)	23 (46.0)	28 (52.8)	23 (32.4)	0.149
Female (%)	36 (51.4)	26 (50.0)	27 (54.0)	25 (47.2)	48 (67.6)	
BMI, kg/m^2^	26.0 ± 3.6	25.5 ± 3.3	24.9 ± 2.4	24.9 ± 3.7	25.2 ± 3.5	0.349
eGFR, ml/min/1.73 m^2^	101.4 ± 10.5	100.2 ± 13.1	98.3 ± 13.2	98.7 ± 14.5	97.5 ± 11.4	0.371
Fasting blood glucose, mg/dL	105.0 ± 31.9	105.5 ± 19.9	101.7 ± 14.7	101.6 ± 8.8	103.1 ± 12.1	0.763
LDL, mg/dL	132.7 ± 31.5	129.7 ± 33.9	133.4 ± 39.4	133.5 ± 33.6	134.1 ± 26.6	0.962
HbA1C, %	5.8 ± 0.9	5.8 ± 0.6	5.6 ± 0.6	5.6 ± 0.4	5.7 ± 0.4	0.137
Spot urine ACR	10.8 ± 7.1	9.8 ± 7.5	7.6 ± 5.2	9.3 ± 6.1	8.7 ± 6.5	0.106
Office SBP	138.2 ± 9.2^a^	139.0 ± 9.1^a^	138.6 ± 7.4^a^	141.5 ± 6.9^a,b^	144.3 ± 10.3^b^	< 0.001
24-h ambulatory SBP	141.8 ± 12.0^a^	136.5 ± 10.1^b^	132.2 ± 8.3^b,c^	130.8 ± 7.4^c^	125.4 ± 10.2^d^	< 0.001
Daytime ambulatory SBP	149.8 ± 11.8^a^	141.5 ± 9.3^b^	136.1 ± 7.3^c^	134.2 ± 6.9^c^	127.7 ± 9.6^d^	< 0.001
Nighttime ambulatory SBP	128.9 ± 15.3^a^	127.9 ± 13.2^a^	124.8 ± 11.5^a,b^	123.6 ± 11.3^a,b^	118.8 ± 13.3^b^	< 0.001

*BMI* Body mass index, *eGFR* Estimated glomerular filtration rate, *LDL* Low density lipoprotein, *ACR* Urine albumin-to-creatinine ratio, *SBP* Systolic blood pressure

^*^p-value given by the analysis of variance, where the different superscript alphabets (a, b, c and d) represent significant differences at an alpha level of 0.05, as given by the post hoc Tukey’s HSD analysis

*p*-value of sex ratio was given by Chi-square test between five groups

In the multivariate analysis adjusted for age, sex, presence of diabetes, and BP (Table 5), the SD and cv of 24-h SBP were higher in the ME > –5 mmHg group than in the ME –5 to < 0 mmHg, Ref, and WE groups. Among patients with ambulatory daytime hypertension, those with ME > –5 mmHg exhibited higher SD values than those in the other groups and a higher cv for 24-h SBP than those in the ME –5 to < 0 mmHg, Ref, and WE 5 to < 10 mmHg groups. However, there were no differences in the cv values between the ME > –5 mmHg and WE ≥ 10 mmHg groups.

**Table 5:** Systolic blood pressure variability among groups categorized by systolic blood pressure difference

	**Systolic blood pressure difference between office and daytime ambulatory blood pressure**					
	**Masked effect**		**Reference**	**White-coat effect**		
	** > -5 mmHg**	**-5 to < 0 mmHg**	**0 to < 5 mmHg**	**5 to < 10 mmHg**	** ≥ 10 mmHg**	p*
All, n	70	52	50	53	71	
SD of 24-h SBP, mmHg	17.5 ± 5.0^a^	14.7 ± 3.2	13.9 ± 3.6	13.9 ± 3.7	13.5 ± 4.0	< 0.001
SD of daytime SBP, mmHg	14.3 ± 5.0	12.7 ± 3.8	12.5 ± 4.7	12.8 ± 4.1	12.9 ± 4.9	0.396
SD of nighttime SBP, mmHg	11.8 ± 4.4	11.3 ± 3.9	10.9 ± 4.4	11.0 ± 4.1	10.3 ± 4.3	0.875
cv of 24-h SBP, %	12.4 ± 3.3^a^	10.8 ± 2.3	10.5 ± 2.9	10.7 ± 2.9	10.8 ± 3.0	0.001
cv of daytime SBP, %	9.5 ± 3.0	9.0 ± 2.6	9.2 ± 3.5	9.5 ± 2.9	10.1 ± 3.6	0.438
cv of nighttime SBP, %	9.2 ± 3.3	8.8 ± 2.7	8.8 ± 3.7	8.8 ± 3.1	8.6 ± 3.3	0.828
ARV of 24-h SBP, mmHg	12.4 ± 4.4	11.0 ± 2.8	10.4 ± 2.9	11.3 ± 3.5	10.8 ± 3.5	0.386
Ambulatory daytime hypertension, n	68	47	43	43	38	
SD of 24-h SBP, mmHg	17.6 ± 5.1^a^	14.7 ± 3.3	13.9 ± 3.8	13.7 ± 3.9	14.2 ± 3.8	0.001
SD of daytime SBP, mmHg	14.3 ± 5.1	12.8 ± 3.9	12.7 ± 4.7	12.4 ± 4.3	13.3 ± 4.8	0.277
SD of nighttime SBP, mmHg	11.8 ± 4.5	11.4 ± 3.9	10.7 ± 4.4	10.9 ± 4.3	10.6 ± 4.1	0.894
cv of 24-h SBP, %	12.3 ± 3.4^b^	10.6 ± 2.3	10.4 ± 3.0	10.4 ± 2.9	10.9 ± 2.8	0.001
cv of daytime SBP, %	9.5 ± 3.0	8.9 ± 2.6	9.2 ± 3.4	9.1 ± 3.0	9.9 ± 3.3	0.280
cv of nighttime SBP, %	9.1 ± 3.3	8.8 ± 2.8	8.5 ± 3.6	8.7 ± 3.2	8.6 ± 2.8	0.882
ARV of 24-h SBP, mmHg	12.5 ± 4.4	11.1 ± 2.8	10.3 ± 2.9	11.0 ± 3.7	11.2 ± 3.6	0.345

*SD* Standard deviation, *cv* Coefficient of variation, *SBP* Systolic blood pressure, *ARV* Average real variability

^*^p-value by ANCOVA adjusted with age, sex, presence of diabetes and ambulatory BP

^a^ *p* < 0.05 compared to masked effect -5 to < 0 mmHg, reference and white-coat effect groups with Bonferroni correction

^b^ *p* < 0.05 compared to masked effect -5 to < 0 mmHg, reference and white-coat effect 5 to < 10 mmHg group with Bonferroni correction

Among the individuals with sustained systolic hypertension, 20.8% and 23.3% were in the ME > –5 mmHg and –5 to < 0 mmHg groups, respectively (Table 6). However, 14.4% and 23.3% were in the WE ≥ 10 mmHg and 5 to < 10 mmHg groups, respectively. In individuals with masked systolic hypertension, 68.5% and 20.4% were in the ME > –5 mmHg and –5 to < 0 mmHg groups, respectively. SBP variabilities did not significantly differ among the systolic hypertension phenotypes.

**Table 6:** Distribution of systolic blood pressure difference and systolic blood pressure variability among systolic hypertension phenotypes

	**Systolic normotension**	**White-coat systolic hypertension**	**Masked systolic hypertension**	**Sustained systolic hypertension**	p*
n	91	31	54	120	
SBP difference					
ME > -5 mmHg, n (%)	8 (8.8)	0 (0.0)	37 (68.5)	25 (20.8)	
ME -5 to < 0 mmHg, n (%)	13 (22)	0 (0.0)	11 (20.4)	28 (23.3)	
Ref 0 to < 5 mmHg, n (%)	22 (24.2)	0 (0.0)	6 (11.1)	22 (18.3)	
WE 5 to < 10 mmHg, n (%)	20 (22.0)	5 (16.1)	0 (0.0)	28 (23.3)	
WE ≥ 10 mmHg, n (%)	28 (30.8)	26 (83.9)	0 (0.0)	17 (14.2)	
SBP variability					
SD of 24-h SBP, mmHg	12.9 ± 3.2	13.9 ± 4.0	16.2 ± 3.8	15.9 ± 4.8	0.156
SD of daytime SBP, mmHg	11.6 ± 3.8	13.4 ± 4.7	13.6 ± 3.8	14.0 ± 5.2	0.283
SD of nighttime SBP, mmHg	10.0 ± 4.5	9.9 ± 3.8	11.6 ± 4.0	11.9 ± 4.1	0.763
cv of 24-h SBP, %	10.4 ± 2.6	11.2 ± 3.2	11.8 ± 2.6	11.2 ± 3.3	0.107
cv of daytime SBP, %	9.2 ± 3.0	10.5 ± 3.6	9.4 ± 2.5	9.5 ± 3.4	0.278
cv of nighttime SBP, %	8.6 ± 3.7	8.6 ± 3.4	9.2 ± 2.9	9.0 ± 3.0	0.773
ARV of 24-h SBP, mmHg	10.4 ± 2.8	10.6 ± 3.4	11.4 ± 3.3	11.9 ± 4.1	0.617

Systolic hypertension phenotypes were classified into systolic normotension (office systolic and daytime ambulatory systolic normotension), white-coat systolic hypertension (office systolic hypertension but daytime ambulatory systolic normotension), masked systolic hypertension (office systolic normotension but daytime ambulatory systolic hypertension) and sustained systolic hypertension (office systolic and daytime ambulatory systolic hypertension)

Systolic blood pressure difference was calculated by subtracting ambulatory daytime SBP from office SBP

^*^p-value by ANCOVA adjusted with age, sex, presence of diabetes and BP

*SD* Standard deviation, *cv* Coefficient of variation, *SBP* Systolic blood pressure, *ARV* Average real variability

## Discussion

In this study, we observed a significant negative and independent association of the magnitude of the SBP difference between office and daytime ambulatory measurements with ambulatory SBP variability in individuals without HMOD. Individuals with a negative SBP difference ≥ –5 mmHg had higher 24-h SBP variability than individuals with a negative SBP difference < –5 mmHg or those with a positive SBP difference. These findings were persistent in individuals with ambulatory daytime hypertension. The SBP difference groups were evenly distributed in individuals with sustained systolic hypertension. There was no significant difference in ambulatory SBP variability among the conventional systolic hypertension phenotypes.

The results of our study suggest that the cardiovascular risk may differ depending on the magnitude of the negative difference between office and ambulatory daytime SBP (masked effect) even in individuals with ambulatory hypertension, and the magnitude of the masked effect may be a better measure for the assessment of cardiovascular risk than the conventional classification of systolic hypertension phenotypes in individuals without apparent HMOD. As shown in the Table 6, 20.8% of patients with sustained systolic hypertension had negative SBP difference > –5 mmHg and 18.3% had negative SBP difference 0 to < 5 mmHg. In the conventional classification of hypertension phenotypes, they may have similar cardiovascular risk. However, if our hypothesis is proven in the further studies, individuals with negative SBP difference > –5 mmHg may have greater cardiovascular risk than those with negative SBP difference 0 to < 5 mmHg. Likewise, the cardiovascular risk may be different between individuals with negative SBP difference > –5 mmHg and 0 to < 5 mmHg even though they have classified as having masked hypertension.

Previous studies have reported that increased BP variability is associated with alterations in the microcirculation [13]. which may comprise an early form of organ damage in patients with hypertension [14]. Microcirculation alterations increase large artery stiffness via increases in mean BP. Increased large artery stiffness elevates central pulse pressure, which in turn damages target organs and small arteries in a vicious cycle [15, 16]. It is plausible that individuals with early microvascular changes may have a higher risk than individuals without such changes. Therefore, our results indicate the possibility of earlier vascular change in individuals with a larger magnitude of masked effect than in those with relatively smaller magnitudes of masked and white-coat effects, even in the absence of other HMODs.

To our knowledge, only one published study (the African American Study of Kidney Disease and Hypertension; AASK Cohort study) has evaluated the implication of the magnitude of the masked effect on patient outcomes [8]. The authors reported a U-shaped association between mortality and the magnitude of the difference between office and ambulatory SBP. The white-coat effect was also associated with a higher risk of death. Consistent with the findings of the AASK Cohort study [8]. individuals with a larger masked effect seemed to have a higher risk in our study. However, high 24-h SBP variability was not observed among individuals exhibiting the white-coat effect. This difference may be explained by differences in the study populations. The AASK study included patients who were on antihypertensive medication and had reduced renal function and long duration of hypertension. The median follow-up duration of the AASK Cohort study was 9.9 years [8]. The cardiovascular risk of the white-coat effect in high-risk patients seemed to increase over time [17], and other studies have reported controversial findings [18]. However, our study was cross-sectional and included participants visiting the outpatient clinic for a first-time diagnosis of hypertension. These patients were not on antihypertensive medications, and the duration of hypertension may have been too short for the development of apparent hypertension-mediated cardiovascular disease.

The strengths of our study include the study population and method of measuring BP. First, antihypertensive medications may change the magnitude of the masked effect and BP variability. Various antihypertensive medications have different effects on BP variability [19]. The participants enrolled in our study were not on antihypertensive medications, allowing us to exclude the influence of antihypertensive medications on BP variability. Second, we evaluated the potential of the masked effect as a cardiovascular risk factor in a low-risk population. The existence of HMOD indicates increased cardiovascular risk and can be associated with increased BP variability. Therefore, we included individuals without apparent HMOD. Third, we obtained research-graded measurements of office and ambulatory BP. Office BP was measured after 5 min of seated rest at 1-min intervals, and measurements were obtained three times at each of three consecutive visits in accordance with the standard method recommended in the guidelines [1, 2]. Individuals with invalid ambulatory BP data were excluded from the analysis.

Nonetheless, our study also had several limitations. First, this was a cross-sectional study. Therefore, the association of the masked effect with long-term outcomes should be investigated by future studies. Second, we used daytime ambulatory BP as a reference rather than 24-h ambulatory BP. Daytime ambulatory BP does not reflect nighttime BP. However, we used daytime ambulatory BP in the calculation of the BP difference between office and ambulatory BP because daytime ambulatory BP may more effectively reflect real office BP, which is measured during the daytime. Third, we did not evaluate arterial stiffness, echocardiographic LVH, the ankle-brachial index, or advanced retinopathy in the assessment of HMOD. However, few patients in our study population were likely to have had these conditions, meaning that the results would not significantly change. Fourth, the clinical relevance of the masked effect on the cardiovascular outcome is uncertain because, to our knowledge, only one published study has reported the implications of its magnitude on mortality. Therefore, it may be premature to assert that the masked effect can be used for risk assessment. Its clinical implications on cardiovascular outcomes should be evaluated by long-term studies with populations at various risk degrees. If the magnitude of the masked effect is demonstrated to be an additional risk factor, the masked effect could be considered in the diagnosis and treatment of patients with hypertension.

## Conclusions

Our study suggests that the magnitude of the negative difference between office and ambulatory daytime SBP (masked effect) may be a potential cardiovascular risk factor even in individuals without apparent HMOD.

## Data Availability

The data presented in this study are available on request from the corresponding author.

## Abbreviations

ACR: Albumin-to-creatinine ratio
ARV: Average real variability
BMI: Body mass index
BP: Blood pressure
CKD: Chronic kidney disease
cv: Coefficient of variation
DBP: Diastolic blood pressure
eGFR: Estimated glomerular filtration rate
FBS: Fasting blood sugar
HMOD: Hypertension-mediated organ damage
LDL: Low-density lipoprotein
LVH: Left ventricular hypertrophy
ME: Masked effect
Ref: Reference
SBP: Systolic blood pressure
SD: Standard deviation
WE: White-coat effect

## References

[1] Williams B, Mancia G, Spiering W, Agabiti Rosei E, Azizi M, Burnier M (2018). 2018 ESC/ESH Guidelines for the management of arterial hypertension. Eur Heart J.

[2] Whelton PK, Carey RM, Aronow WS, Casey DE, Collins KJ, Dennison Himmelfarb C (2018). 2017 ACC/AHA/AAPA/ABC/ACPM/AGS/APhA/ASH/ASPC/NMA/PCNA guideline for the prevention, detection, evaluation, and management of high blood pressure in adults: executive summary: a report of the American college of cardiology/american heart association task force on clinical practice guidelines. Hypertension.

[3] Lewington S, Clarke R, Qizilbash N, Peto R, Collins R (2002). Prospective Studies Collaboration. Age-specific relevance of usual blood pressure to vascular mortality: a meta-analysis of individual data for one million adults in 61 prospective studies. Lancet..

[4] Rapsomaniki E, Timmis A, George J, Pujades-Rodriguez M, Shah AD, Denaxas S (2014). Blood pressure and incidence of twelve cardiovascular diseases: lifetime risks, healthy life-years lost, and age-specific associations in 1.25 million people. Lancet..

[5] Anstey DE, Pugliese D, Abdalla M, Bello NA, Givens R, Shimbo D (2017). An update on masked hypertension. Curr Hypertens Rep.

[6] Kim HC, Ihm SH, Kim GH, Kim JH, Kim KI, Lee HY (2019). 2018 Korean society of hypertension guidelines for the management of hypertension: part I-epidemiology of hypertension. Clin Hypertens.

[7] Lee HY, Shin J, Kim GH, Park S, Ihm SH, Kim HC (2019). 2018 Korean society of hypertension guidelines for the management of hypertension: part II-diagnosis and treatment of hypertension. Clin Hypertens.

[8] Ku E, Hsu RK, Tuot DS, Bae SR, Lipkowitz MS, Smogorzewski MJ (2019). Magnitude of the difference between clinic and ambulatory blood pressures and risk of adverse outcomes in patients with chronic kidney disease. J Am Heart Assoc.

[9] Kim JS, Rhee MY, Kim CH, Kim YR, Do U, Kim JH (2021). Algorithm for diagnosing hypertension using out-of-office blood pressure measurements. J Clin Hypertens (Greenwich).

[10] Kim CH, Kim JS, Rhee MY (2020). Characteristics of Individuals with disagreement between home and ambulatory blood pressure measurements for the diagnosis of hypertension. Healthcare (Basel).

[11] Levey AS, Stevens LA, Schmid CH, Zhang YL, Castro AF, Feldman HI (2009). A new equation to estimate glomerular filtration rate. Ann Intern Med.

[12] Hansen TW, Thijs L, Li Y, Boggia J, Kikuya M, Bjorklund-Bodegard K (2010). Prognostic value of reading-to-reading blood pressure variability over 24 hours in 8938 subjects from 11 populations. Hypertension.

[13] Rizzoni D, Muiesan ML, Montani G, Zulli R, Calebich S, Agabiti-Rosei E (1992). Relationship between initial cardiovascular structural changes and daytime and nighttime blood pressure monitoring. Am J Hypertens.

[14] Schiffrin EL (2004). Remodeling of resistance arteries in essential hypertension and effects of antihypertensive treatment. Am J Hypertens.

[15] Laurent S, Briet M, Boutouyrie P (2009). Large and small artery cross-talk and recent morbidity-mortality trials in hypertension. Hypertension.

[16] Rosei EA, Chiarini G, Rizzoni D (2020). How important is blood pressure variability?. Eur Heart J Suppl.

[17] Verdecchia P, Reboldi GP, Angeli F, Schillaci G, Schwartz JE, Pickering TG (2005). Short- and long-term incidence of stroke in white-coat hypertension. Hypertension.

[18] Cohen JB, Lotito MJ, Trivedi UK, Denker MG, Cohen DL, Townsend RR (2019). Cardiovascular events and mortality in white coat hypertension: a systematic review and meta-analysis. Ann Intern Med.

[19] Rothwell PM, Howard SC, Dolan E, O'Brien E, Dobson JE, Dahlof B (2010). Effects of beta blockers and calcium-channel blockers on within-individual variability in blood pressure and risk of stroke. Lancet Neurol.

